# Supplementary material for: Pd/Cu Single Atom Alloys for Selective Alcohol Dehydrogenation: From Single Crystalline to Nanostructured Model Catalysts
Source: Angew Chem Int Ed Engl. 2025 Dec 18;65(5):e21885. doi: 10.1002/anie.202521885 (PMC12851000; doi:10.1002/anie.202521885)
Supplement: Supplementary file 1 — Supporting information [file ANIE-65-e21885-s001.pdf]

Supporting information for the manuscript:

Pd/Cu single atom alloys for catalysis: from single crystalline to nanostructured model systems for highly selective butanol dehydrogenation

Philipp A. Fredersdorff[1], Jan Smyczek[1], Carsten Schröder[1], Paul Fröhlich[1], Paul Kohlmorgen[1], Stephan Appelfeller[2], Konstantin Neyman[3]* and Swetlana Schauermann[1,4]*

[1]*Institute of Physical Chemistry, Christian-Albrechts-University Kiel, Max-Eyth-Str. 1, 24118 Kiel, Germany.*

[2]*MAX IV Laboratory, Lund University, 22100 Lund, Sweden*

[3] *Departament de Ciència de Materials i Química Física & Institut de Química Teòrica i Computacional (IQTC-UB), Universitat de Barcelona, Barcelona 08028, Spain, ICREA (Institució Catalana de Recerca i Estudis Avançats), Barcelona 08010, Spain*

[4]*Kiel Nano, Surface and Interface Science, Christian-Albrechts-University Kiel, 24118 Kiel, Germany.*

*schauermann@pctc.uni-kiel.de

*konstantin.neyman@icrea.cat

1. Experimental section and computational details

UHV Experiments

All experiments were carried out in two separate ultrahigh vacuum (UHV) systems: an IRAS/molecular beam setup and an STM setup, each equipped with a dedicated preparation chamber. All chambers were maintained at a base pressure below 4.0×10^{-10} mbar at room temperature. The preparation chamber was isolated from the respective analysis chambers by a UHV gate valve.

The Cu(111) single crystal (MaTeck GmbH; 10 × 10 mm in the IRAS setup, 5 mm diameter in the STM setup) was cleaned via repeated cycles of Ar^+ ion bombardment at room temperature (~7 µA) followed by annealing to 800 K. The NiAl(110) single crystal (MaTeck GmbH; 10 × 10 mm in the IRAS setup, 5 mm diameter in the STM setup) was similarly cleaned by repeated Ar^+ sputtering (1×10^{-5} mbar Ar, 1.25 keV, ~13 µA) at room temperature, followed by annealing to 1150 K. The Al_2O_3/NiAl(110) surface was prepared by oxidizing the NiAl(110) substrate in 2×10^{-6} mbar O_2 at 620 K for 10 min, followed by annealing to 1100 K. The long-range order and cleanliness of the resulting Al_2O_3 film were confirmed using low-energy electron diffraction (LEED), Auger electron spectroscopy (AES), scanning tunneling microscopy (STM), and IRAS of adsorbed CO. No CO signal was detected on the clean alumina surface at 100 K. The prepared Al_2O_3/NiAl(110) film is shown in Figure S1.

Butanol (Sigma-Aldrich, 99.5%) was purified by multiple freeze–pump–thaw cycles prior to use. Pd and was deposited from a Pd rod and Cu was deposited out of a Mo crucible using a Focus EFM evaporator. Deposition rates were calibrated with a quartz crystal microbalance.

For in situ IRAS measurements, two doubly differentially pumped effusive molecular beams were used for molecular dosing, with typical fluxes of 3.5×10^{12} molecules cm^{-2} s^{-1} for butanol. IRAS spectra were recorded with a vacuum FT-IR spectrometer (Bruker Vertex 80v) equipped with a mercury–cadmium–telluride (MCT) detector at a spectral resolution of 2 cm^{-1}. Further technical details are provided elsewhere.[1]

STM measurements were performed in a separate UHV chamber (base pressure <2 × 10^{-10} mbar) using a variable-temperature Aarhus 150 SPM (SPECS Surface Nano Analysis GmbH), operating between 115–300 K. Gas exposure was controlled via dedicated dosing lines. All measurements were carried out in a constant current mode, and the bias voltage U_B was applied to the sample, which is specified in the figure caption in mV. The W-tip, supplied by SPECS, was sharpened in situ by repeated Ar^+ sputtering (1.25 keV, 5 × 10^{-6} mbar, 5 min). Tip sharpness was verified on pristine Cu(111) surfaces. STM calibration was performed by imaging Cu(111) and highly oriented pyrolytic graphite HOPG(0001) reference surfaces with known interatomic distances (d_{Cu-Cu} = 2.55 Å; d_{C-C} = 2.456 Å).[2, 3]

DFT Calculations

Spin-restricted DFT calculations were performed using the plane-wave code.[4 5] The generalized-gradient exchange-correlation functional by Perdew, Becke and Ernzerhof (PBE)[6] was employed in combination with the projector augmented wave (PAW) representation of core electrons.[7, 8] The cutoff energy for the plane-wave functions was set to 415 eV. One-electron Kohn-Sham energy levels were smeared by 0.1 eV and the total energies converged to 0.5×10^{-6} eV were extrapolated to zero smearing. Atoms included in the geometry optimization were locally displaced without any restrictions until the forces acting on each of these atoms decreased below 0.2 eV/nm.

Two types of structural models were used. Single crystal (111) surfaces of Cu, Pd and Pd/Cu SAA were represented by slab models containing 6 atomic layers, in which atoms of 4 upper layers were allowed to relax, while atomic positions of the other two layers were kept fixed the same as in the bulk of the corresponding metals. The surface cells ranged from 2×2 to 4×4, containing 24 to 96 atoms per slab supercell, respectively. The smallest surface cells were calculated using 5×5 surface k-points, whereas the 3×3 surface k-points were used for the largest surface cells. Representative nanoparticle models of Cu, Pd and Pd/Cu SAA correspond to 328-atomic particles cut from the fcc bulk metal structures, in which all atoms were allowed to relax during full structure optimization calculations. These ca. 2.1 nm large nanoparticles were placed in the 3.2 × 3.2 × 3.2 nm^3 large cubic cells with enough space between adjacent periodically repeated particles making their interactions negligible.[9] The Brillouin zone for nanoparticle models was sampled only at the Γ-point. In all studied Pd/Cu SAA models single Pd atoms were located in surface positions. CO vibrational frequencies for the molecules adsorbed in various positions were calculated numerically by displacements of the C and O atoms by 0.02 Å in each Cartesian direction.

2. Full experimental details for Figures 1 – 5 of the main manuscript

Figure 1: Infrared transmission (IR) spectra following CO saturation on (a) single crystalline and (b) nanostructured SAA Pd/Cu model catalysts: (1a) Cu(111) at 100 K, (1b) Cu(111) at 230 K, (1c) Pd(111) at 230 K, (1d) as-deposited 0.3 ML Pd/Cu(111) deposited at 300 K and measured at 230 K, (1e) 0.3 ML Pd/Cu(111) deposited at 300 K and after annealing to 500 K, measured at 230 K, (1f) 0.3 ML Pd/Cu(111) deposited at 300 K and after annealing to 600 K, measured at 230 K, (1g) 0.3 ML Pd/Cu(111) deposited at 300 K and after annealing to 700 K, measured at 230 K and (1h) 0.3 ML Pd/Cu(111) deposited at 300 K and after annealing to 800 K, measured at 230 K. Part (b) of the Figure shows CO saturation on (2) Al_2O_3/NiAl(110) at 100 K, (2a) 1 ML Cu nanoparticles (NPs) on Al_2O_3/NiAl(110) after deposition at 300 K and annealing to 500 K, measured at 100 K with increasing CO exposure until saturation, (2b) 1 ML Cu NPs/Al_2O_3/NiAl(110) measured at 230 K, (2c) 0.05 ML Pd/Al_2O_3/NiAl(110) deposited at 300 K and measured at 230 K, and (2d) 0.05 ML Pd/Al_2O_3/NiAl(110) annealed to 500 K, measured at 230 K, (2e) as-

deposited 0.05 ML Pd/1 ML Cu NPs/Al_2O_3/NiAl(110) at 300 K and measured at 230 K, (2f) 0.05 ML Pd/1 ML Cu NPs/Al_2O_3/NiAl(110) deposited at 300 K and after annealing to 500 K, measured at 230 K. Schematics alongside each spectrum illustrate simplified representations of the surface under CO saturation conditions.

Figure 2: Scanning tunneling microscopy (STM) images of (a) Pd single-atoms on Cu(111) formed after annealing to 550 K and (b) pristine Al_2O_3/NiAl(110). The bottom row presents an overview and a high-resolution STM image of Cu nanoparticles on Al_2O_3/NiAl(110) in (c) and (d), Pd deposited onto Cu-NPs/Al_2O_3/NiAl(110) at 300 K in (e) and (f) and Pd/Cu-NPs/Al_2O_3/NiAl(110) after annealing to 550 K in (g) and (h). Tunneling conditions: (a) (15.9×15.9 nm^2, 2024 mV, 0.18 nA), (b) (33.2×33.2 nm^2, -4101 mV, 0.52 nA), (c) (87.0×87.0 nm^2, 5084 mV, 0.18 nA), (d) (27.5×27.5 nm^2, 5084 mV, 0.18 nA), (e) (87.0×87.0 nm^2, 5084 mV, 0.13 nA), (f) (27.5×27.5 nm^2, 5084 mV, 0.14 nA), (g) (87.0×87.0 nm^2, 5084 mV, 0.13 nA), (h) (27.5×27.5 nm^2, 5084 mV, 0.13 nA)

Figure 3. (a) Pd_2Cu_{326} nanoparticle with two single Pd atoms located in the edge (CN=6) and the (111) facet (CN=9) positions representing the calculated 328-atom nanoparticle exposing various Pd_1 surface sites. (b) Cu(111), Pd(111), and Pd_1Cu(111) single-crystal surfaces with CO adsorbed in the atop positions. Optimized CO adsorption geometries on the (111) facet (c) (CN=9) and corner (d) (CN=6) sites of Cu_{328}, Pd_{328}, and Pd_1Cu_{327} nanoparticles. Insets show experimental and theoretical CO stretching frequencies, metal–carbon r(M–C) bond distances, average metal–metal r(M–M) distances for the metal atoms bonded to CO, and partial Bader charges of the same atoms.

Figure 4. Reactivity of model catalysts in butanol dehydrogenation proceeding according to competing reaction pathways shown in (a). Formation rates (arbitrary units) of butanal (blue, multiplied by 3) and CO (red) obtained over Pd(111) (b), Cu(111) (c), and SAA-based catalysts: Pd (0.3 ML)/Cu(111) – “as deposited” (d) and annealed to 550 K (d); as well as Pd (0.3 ML)/Cu-NPs (1 ML)/Al_2O_3 – “as deposited” (f) and annealed to 550 K (g). All TPD traces were detected after exposure of 0.8 L butanol at 180 K, where the mass traces at m/z = 28 and 72 (multiplied by 3 for visualization) have been followed for the decomposition and dehydrogenation pathway, respectively.

Figure 5. Butanol yield (a and b), CO yield (c and d) and calculated butanal selectivity derived from TPD data (e and f) depending on the Pd loading from 0 to 0.55 ML Pd on Cu(111) as a host (left, a, c and e) and Cu-NPs (1 ML)/ Al_2O_3 as a host (right, b, d and f) with the respective surface as deposited (black and gray) and annealed (blue and purple).

3. Supporting STM Images

Figure S1 presents scanning tunneling microscopy (STM) images (a) and the LEED pattern (b) of the pristine Al_2O_3 thin film grown on NiAl(110) by the procedure described above. The large-scale STM image in Figure S1 (a) confirms the formation of a well-ordered Al_2O_3 film.[10, 11] The inset displays a high-resolution image. These results confirm both the crystallinity and the cleanliness of the support prior to nanoparticle deposition as previously described in detail.[10]

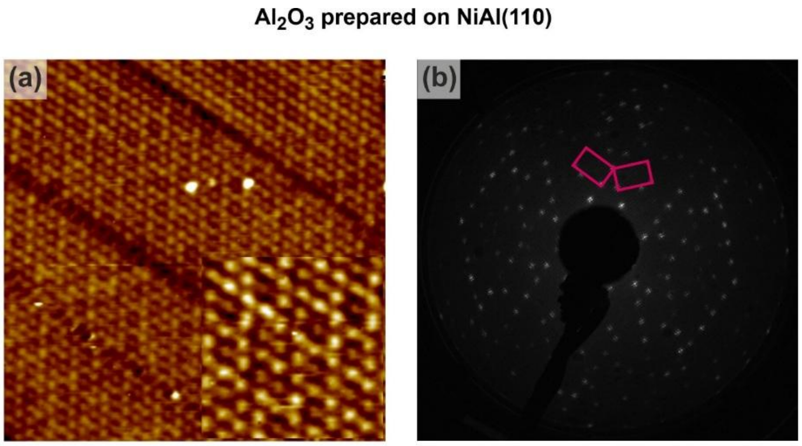

Figure S1: (a) STM image of pristine Al_2O_3 grown on NiAl(110) (33.2×33.2 nm^2, -4101 mV, 0.52 nA) and (b) its LEED pattern at 84 eV.

Figure S2 tracks the thermal evolution of 0.15 ML of Pd deposited on Cu(111). In the as-deposited state (a and b), Pd forms small clusters, preferentially decorating the step edges of the Cu terraces. Upon annealing to 550 K for 2 minutes (c and d), these clusters disperse into isolated Pd atoms, clearly visible as brighter features on the terraces and step edges (d). This transformation is consistent with the formation of Pd single atoms alloyed into Cu(111) as reported earlier for this system.[12-14]

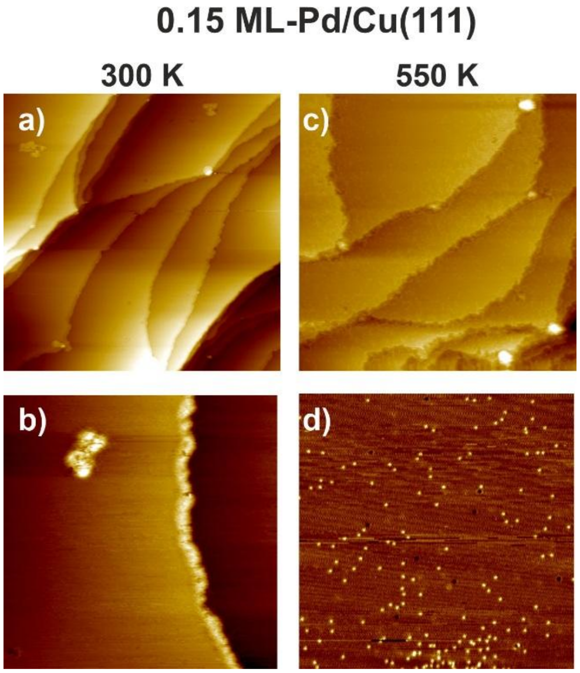

Figure S2: Large scale and zoom in STM images of 0.15 ML Pd deposited onto Cu(111) a) (200×200 nm^2, 1000 mV, 0.1 nA) and b) (58.7×58.7 nm^2, 1000 mV, 0.1 nA)) deposited at 300 K and c) (100×100 nm^2, -2000 mV, 0.1 nA) and d) (45.6×45.6 nm^2, -2000 mV, 0.1 nA) after annealing to 550 K for 2 min.

4. Literature assignments of CO frequencies on Cu, Pd and Pd/Cu-SAA

Table S1 provides an overview of previously reported CO vibrational frequencies on Cu and Pd single crystals as well as on nanoparticles. In addition, it summarizes published CO IRAS frequencies for CO adsorbed on doped single-atom alloy (SAA) centers. This compilation serves as a valuable reference for the assignment of CO vibrational features discussed in the main manuscript for Pd/Cu(111) and Pd/Cu–NP/Al_2O_3.

Table S1: Vibrational frequencies (in cm^{-1}) of CO adsorbed on Cu single crystals, Cu nanoparticles, Pd nanoparticles and Single Atom Alloys on Cu(111) previously reported in literature.

Surface	On top	Bridge	Three-fold hollow
Cu(111)[15-17]	2070–2080		
Cu(110)[15, 18, 19]	2088–2104		
sputtered Cu(111) [19]	2099; 2071		
Cu/SiO_2/Mo(110)[19, 20]	2106		
2.2nmPd/Al_2O_3/NiAl(110)[21]	2103	1986	
Pd(5ML)/Al_2O_3/Mo(100)[22]	2074-2108	1894-1996	1835-1887
Pd/Cu(110)[23]	2041–2117	1990–1990	
Cu(111), 0.03 ML Pd/Cu(111), 10 L at 100 K[24]	2068	1833, 1816	
0.14 ML Pd/Cu(111)10 L at 300 K[24]	2064		
>0.23 ML Pd/Cu(111) 1×10^{-2} Torr at 300 K[24]	2073	1925	1890
NiCu(111)[25]	2004	1874	
RhCu(111)[26]	2007	1899	
PdAg(111)[27]	2047-2062	1955	
TiCu(111)[28]	2050		
PtCu(111)[29]	2046-2041		
Pt/Cu(211)[30]	2045-2043		

5. Supporting DFT Results

Table S2 presents the results of DFT calculations for CO adsorption on various calculated model systems, including Cu(111), Pd(111), Pd_1/Cu(111), and 328-atom nanoparticles composed of pure Cu, pure Pd, and bimetallic Pd_5Cu_{323} species. In the nanoparticles, the Pd adsorption sites are further differentiated by their local geometry and coordination number (CN): sites on (111) and (100) terraces (CN=9 and CN=8, respectively), edge sites (CN=7), and corner sites (CN=6). The calculated dataset for CO adsorption complexes formed on top of metal atoms X includes CO adsorption energies E_{ad}, vibrational stretching frequencies $\nu(CO)_{theo}$ compared to the experimental values $\nu(CO)_{exp}$, metal–CO distances X–CO, intramolecular C–O distances, Bader charges (q), and average metal–metal distances in the first coordination environment of the atom X, $(X–M)^{ave}$.

These parameters together allow a detailed analysis of how the local electronic and geometric environment of the adsorption site, especially in Pd_1/Cu single-atom alloy (SAA) configurations, influences CO binding properties. Notably, the CO stretching frequency for Pd_1/Cu(111) shows a non-linear redshift that lies below both Cu(111) and Pd(111) values, a deviation not explained by simple linear interpolation between parent metals.

This non-linearity is mirrored in the longer X–CO bond distance in the Pd_1/Cu(111) system than in either of the monometallic Cu(111) and Pd(111) systems. While differences in atomic radii (Cu: 1.28 Å, Pd: 1.37 Å) can partly account for this trend, the analysis suggests that charge redistribution between Pd and Cu plays a significant role. Bader charge analysis indicates that single Pd atoms in Cu environments acquire negative charge (~ –0.3 |e|) from the more noble Cu host. This electron gain likely weakens the Pd–CO interaction, leading to the bond elongation and redshifted CO vibrational frequency.

Notably, across CN=6 to CN=9, Pd_1 sites in Pd_5Cu_{323} NPs consistently show longer M–CO distances and lower CO stretch frequencies compared to their monometallic analogues, while maintaining intermediate adsorption energies.

This table supports the broader conclusions of the manuscript, showing that single-atom alloying induces unique electronic and geometric modifications, which in turn yield distinct CO vibrational signatures and binding properties. These trends are critical for understanding the catalytic function of SAAs and the evolution of active sites during thermal treatment or reaction conditions.

Table S2: Calculated DFT data for atop CO adsorption on Cu(111), Pd(111), Pd_1/Cu(111) single-crystal models as well as monometallic Cu_{328} and Pd_{328} nanoparticles and bimetallic Pd_5Cu_{323} nanoparticle exposing single Pd atoms in the (111) facet (coordination number CN=9), (100) facet (CN=8), at the edge between (100) and (111) facet (CN=7), at the edge between (111) and (111) facet (CN=7) and at the corner (CN=6) of 328-atomic nanoparticles. CO adsorption energy E_{ad}(CO), vibrational CO frequency $\nu(CO)_{theo}$ compared to the experimental CO frequency $\nu(CO)_{exp}$, distance between CO and metal atom X on which it is adsorbed X-CO, C-O bond distance, Bader charge q of the metal atom X and the average neighbor distance of the atom X with its nearest-neighbor atoms M $(X\text{-}M)^{ave}$.

Model	Site CN	E_{ad}(CO) eV	ν(CO)$_{theo}$ cm^{-1}	ν(CO)$_{exp}$ cm^{-1}	X-CO Å	C-O Å	q |e|	(X-M)^{ave} Å
Single crystal								
$(CO)_1Cu(111)3\times3\times6$	9	-0.81	2015	2072	1.847	1.158	-0.02	2.59
$(CO)_3Cu(111)3\times3\times6$	9	-0.77	2038^{all_sym} 2004^{single}		1.849	1.158	-0.02	2.59
$(CO)_3Pd(111)2\times2\times6$	9	-1.27^{a}	2078^{all_top}	2088	1.897	1.157^{a}	-0.03	2.77
$(CO)_1Pd_1/Cu(111)4\times4\times6$	9	-1.03	2026	2060	1.925	1.157	-0.36	2.61
Nanoparticle								
$(CO)_5Cu_{328}$	6	-1.00	2032^{all}	2106	1.837	1.155	-0.08	2.54
	7 (100/111)	-0.94	2012^{all}		1.844	1.157	-0.03	2.56
	7 (111/111)	-0.89	2019^{all}		1.843	1.156	-0.03	2.56
	8 (100)	-0.81	2005^{all}	2092	1.848	1.158	-0.06	2.60
	9 (111)	-0.68	1994^{all}	2071	1.856	1.158	0.01	2.58
$(CO)_5Pd_{328}$	6	-1.61	2012^{all}	2097	1.873	1.160	-0.08	2.72
	7 (100/111)	-1.55	2014^{all}		1.872	1.160	-0.01	2.75
	7 (111/111)	-1.59	2013^{all}		1.871	1.160	-0.02	2.74
	8 (100)	-1.43	2015^{all}		1.873	1.159	0.01	2.78
	9 (111)	-1.34	2020^{all}		1.871	1.160	0.03	2.77
$(CO)_5Pd_5Cu_{323}$	6	-1.29	2003^{all}	2058	1.920	1.159	-0.28	2.58
	7 (100/111)	-1.33	2005^{all}		1.918	1.160	-0.29	2.60
	7 (111/111)	-1.19	2012^{all}		1.918	1.160	-0.33	2.60
	8 (100)	-1.27	2004^{all}		1.919	1.159	-0.26	2.63
	9 (111)	-1.08	2014^{all}		1.918	1.158	-0.34	2.60

a) For atop $(CO)_1Pd(111)2\times2\times6$.

Figure S3 and Table S3 underline the trend of the significant uplift of the Pd_1 site above the (111) or nanoparticles plane. While CO induces a slight Cu_1 and Pd_1 lift in the monometallic Cu(111), Pd(111), Cu NP and Pd NP of around 0.1 Å for the (111) surfaces and around 0.2 Å on the nanoparticles the rise of the Pd atoms twice as high for the Pd single atom on Cu(111) and the (111) facet of the nanoparticles. In all investigated Pd/Cu systems, Pd_1 atoms with adsorbed CO are lifted by 0.05–0.24 Å above the Cu surface plane due to their larger atomic radius. This effect is particularly pronounced for Pd in the rigid Cu(111) terrace environment, where strain can be relieved primarily by lifting the Pd atom above the plane. By contrast, at the nanoparticle corner sites, where the lattice is more flexible, strain release occurs not only through modest vertical displacement (~0.1 Å, similar to pure Cu and Pd systems) but also by increasing

the lateral Pd–Cu distances. These observations suggest that the Pd uplift is strongly site-dependent, reflecting the balance between lattice rigidity and local geometric flexibility.

Our computational results also indicated that Pd/Cu alloys undergo substantial structural changes as compared to the pure metals – both for single crystalline and nanostructured materials. Thus, Pd atoms in the Cu host experience compressive strain of around –5%, with average Pd–M (M = Cu or Pd) bond distances shortened to 2.58–2.61 Å in the alloy, compared to 2.72–2.77 Å in bulk Pd or Pd nanoparticles. On the other side, the Pd-Cu distance was computed to be longer in the Pd/Cu alloy as compared to the Cu-Cu distance 2.54 – 2.59 Å in bulk Cu or Cu nanoparticles. Notably, the Pd–CO bond length computed for Pd/Cu is systematically increased by ~0.04 Å relative to that in pure Pd, independent of the local coordination of the embedded Pd atom. Interestingly, this M–CO bond length is increased compared to both monometallic Cu and monometallic Pd.

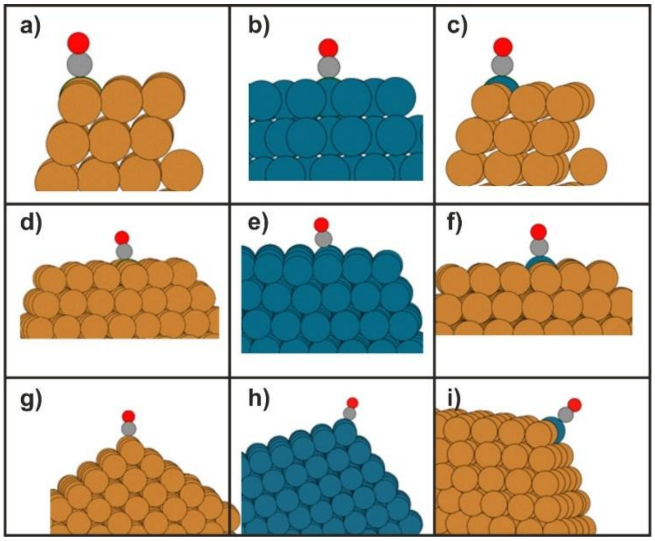

Figure S3: Visualization of the calculated atop CO adsorbed on Cu(111) a), Pd(111) in b), on $Pd_1Cu(111)$(c), CO-Cu_{328}(111) d), CO-Pd_{328}(111) e), CO-Pd_1Cu_{327}(111) f), CO-Cu_{328}(edge) g), CO-Pd_{328}(edge) h) and CO-Pd_1Cu_{327}(edge) in i). The corresponding height above the surface plane of the metal atom where CO is adsorbed on is given in Table SI 3.

Table S3: DFT results of the hight of the OC-**M** metal atom above the surface plane as shown in Figure S3.

surface	height above surface plane / Å
CO-Cu(111)	0.11
CO-Pd(111)	0.10
CO-Pd_1Cu(111)	0.28
CO-Cu_{328}(111)	0.15
CO-Pd_{328}(111)	0.22
CO-Pd_1Cu_{327}(111)	0.46
CO-Cu_{328}(edge)	0.05
CO-Pd_{328}(edge)	0.12
CO-Pd_1Cu_{327}(edge)	0.10

6. In-situ IRAS of butanol on supported Pd/Cu SAA

To monitor the evolution of surface-adsorbed reaction intermediates, we performed in situ IRAS studies under reaction conditions (270 K) during butanol exposure. The corresponding spectra for Cu(111), Pd(111), and annealed Pd/Cu-NPs/Al_2O_3 are shown in Figure S4, together with the spectrum of butanol recorded at 100 K at multilayer coverage, which serves as a reference for the unperturbed butanol molecule. For this reference spectrum, the most dominant and important vibrational modes are the OH stretching vibration at 3252 cm^{-1}, the CH stretching vibrations (ν(CH_x)) between 2970 and 2876 cm^{-1}, the asymmetric and symmetric CH_3 deformation vibrations at 1465 ($\delta_{as}(CH_3)$) and 1379 cm^{-1} ($\delta_s(CH_3)$), the OH deformation vibration at 1250 cm^{-1} (δ_{as}(OH)), the C–C stretching vibration at 1076 cm^{-1} (ν(CC)), and the combined C–O/C–C as well as C–C/C–O stretching vibrations at 1031 (ν(CO/CC)) and 953 cm^{-1} (ν(CO/CC)), respectively. At 270 K, no vibrational features were detected on pristine Cu(111), in line with the lack of reactivity of this surface toward butanol dissociation. In contrast, on Pd(111) butanol adsorption at 270 K gives rise to a new vibrational band at 1810 cm^{-1}.

The 1800 cm^{-1} band is attributed to a C=O-containing decomposition intermediate, most likely a partially hydrogenated carbonyl species (H_xC=O), distinct from adsorption features of butanal[31, 32] at 1726 cm^{-1} or CO between 1850 – 2110 cm^{-1} on Pd(111).[31-33] This intermediate is likely formed during incomplete butanol decomposition to CO. Importantly, this band cannot be attributed to molecular CO, as CO adsorbed on Pd(111) exhibits vibrational bands above 1850 cm^{-1}.[33, 34]

Most importantly, distinct molecular vibrational frequencies are detected on Pd/Cu-NP/Al_2O_3 at 270 K. At this temperature, monolayers of butanol have already desorbed from Al_2O_3 and Cu-NP/Al_2O_3 at 223 and 235 K, respectively. Therefore, the observed vibrations must originate from a surface intermediate bound to Pd sites. Notably, the OH vibrations at 3252 and 1250 cm^{-1} are absent at 270 K, indicating cleavage of

the O–H bond, which is a characteristic signature of alkoxy formation on metal surfaces.[35-37] Furthermore, the combined C–O and C–C stretching modes at 1031 and 964 cm^{-1} are shifted compared to the unperturbed butanol molecule in Figure S4a, while the CH stretching and deformation frequencies remain unchanged. Taken together, these observations provide evidence for a species that is not present in molecular butanol and can be assigned to the butoxy intermediate formed upon hydroxy-H abstraction only on Pd sites. With this, the direct detection of the key reaction intermediate – the butoxy species – on Pd/Cu single-atom alloy nanoparticles is achieved, whereas it does not form on either Cu(111) or Pd(111) surfaces.

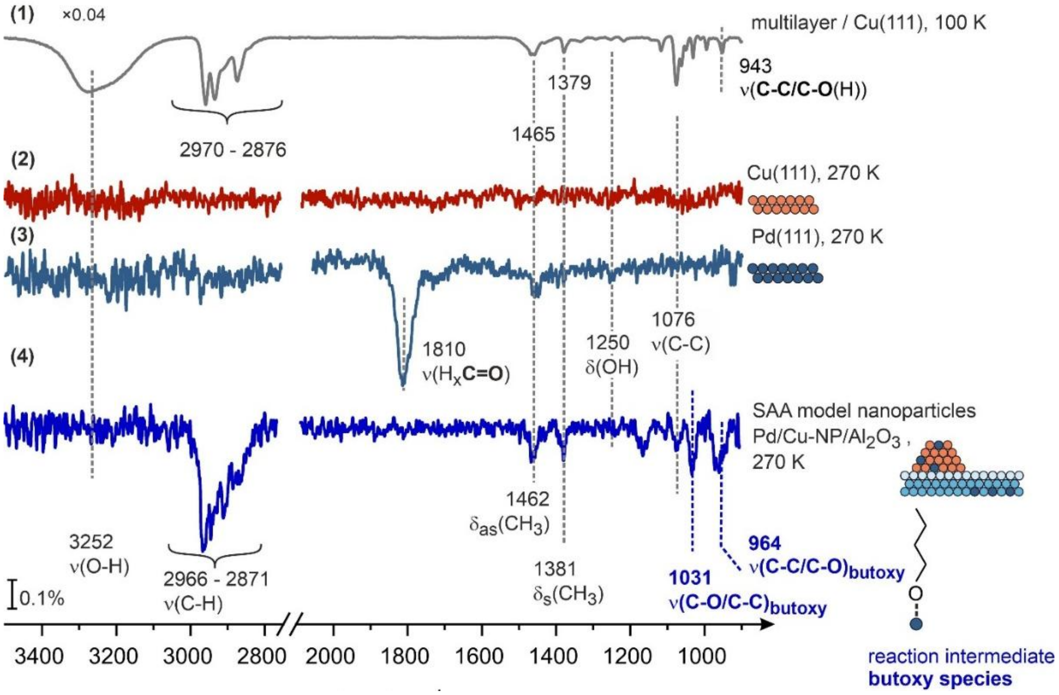

Figure S4. IR Spectra of (1) multilayer butanol adsorbed at 100 K on Cu(111), (2) butanol exposed to Cu(111) at 270 K, (3) butanol exposed to Pd(111) at 270 K and (4) butanol exposed to annealed 0.3 ML-Pd/Cu-NP/Al_2O_3/NiAl(110) at 270 K. The total butanol exposure in b, c and d amounts to $8.4\cdot10^{14}$ molecules/cm^2.

7. Stability of Pd/Cu-NPs SAA model catalyst: distribution of Pd before and after the reaction.

Stability of Pd (0.01 ML)/Cu-NPs/Al_2O_3 model catalyst was addressed by probing the distribution of different adsorption site via CO adsorption before and after the reaction. Figure S5 shows the IR spectrum obtained after saturation of the catalyst with CO before the reaction (spectrum 1) and after the completion of one TPD run (spectrum 2). The TPD was performed in the same way as described in the main text of the manuscript following adsorption of 0.8 L of butanol. Prior the reaction, the spectrum exhibits a single absorption band at 2058 cm^{-1} assigned to isolated Pd atoms. The absence of the peaks related to the bridge or hollow sites (around 1920 cm^{-1}) indicates that only isolated Pd atoms are present on the Cu nanoparticles. After the reaction (spectrum 2), the spectrum looks very similar – there is only one band at 2058 cm^{-1}, confirming that the isolated Pd atoms are preserved on the surface after the TPD run, while no vibrational bands were detected in the range of bridge- and hollow-bonded CO, suggesting that no agglomeration of Pd has occurred. The intensity of the band at 2058 cm^{-1} appears somewhat lower than before reaction. Taking into account the fact that no adsorbate-induced formation of Pd ensembles was observed, the

diminishing of the band at 2058 cm^{-1} can be most likely related to partial diffusion of Pd into subsurface region or site poisoning. Further experimental work is required to address the stability of the isolated sites and their possible unwanted agglomeration during the reaction.

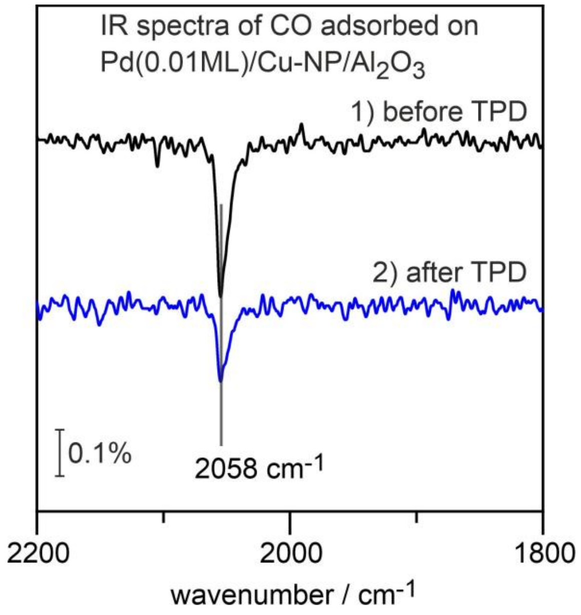

Figure S5. IR spectra obtained after saturation of the Pd(0.01 ML)/Cu-NPs/Al_2O_3 with CO at 230 K prior the reaction (1) and after one TPD run (2). The TPD was carried out after adsorption of 0.8 L of butanol.

8. Possible reasons for the observed differences in the reactivity between Pd/Cu-NPs and Pd/Cu(111).

Thus, the SAA-based catalysis consisting of Pd atoms embedded in Cu-NPs show an outstanding catalytic performance both in terms of the absolute amount of formed butanal and the selectivity as well as maintain these catalytic properties over large range of Pd coverages. In contrast, the single crystalline Pd/Cu(111) catalysts demonstrate quickly declining selectivity with growing Pd content and lower absolute activity. These observations must be related to the nanoscopic nature of Cu-NPs, in which Pd can be embedded not only into the regular Cu(111) terraces but also at the low-coordinated sites, such as edges, corners and their immediate surrounding, which can potentially exhibit higher catalytic activity and ability to stabilize isolated Pd atoms. Generally, we believe that Cu-NPs are most likely able to prevent Pd agglomeration even at relatively high Pd loadings – the property that is lacking in single-crystalline Pd/Cu systems. This conclusion is drawn from the observation that over Pd/Cu-NPs no CO is formed, which is the product resulting from multiple consecutive dissociating steps of butanol and requiring ensembles of agglomerated Pd atoms.

There are two most obvious reasons for the excellent ability of Cu-NPs to keep the Pd atoms in the dispersed form: **(1)** our relatively small Cu-NPs contain a large fraction of low-coordinated sites such as edges, corners and their immediate surrounding, at which isolated Pd atoms have a smaller number of neighboring Cu atoms as compared to the regular (111) facets. It might be possible, that if several Pd atoms reside at these low-coordinated sites, it is energetically more favorable for them to be dispersed as single atoms than to build ensembles. To address this question, we plan to perform computational work, which needs to be

performed for **multiple Pd atoms situated in close vicinity** for two systems – Pd integrated into the low-coordinated sites of Cu-NPs vs. Pd embedded into Cu(111) facets. In this case we are aiming to compare the total energies for two boundary cases – all Pd atoms are isolated vs. all Pd atoms are agglomerated into Pd ensembles – which will allow us to answer the question whether the low-coordinated sites at Cu-NPs can more efficiently prevent Pd agglomeration as compared to the extended Cu(111) facets. **(2)** The second possible reason, which appears feasible taking into account the partial negative charge of isolated Pd atoms, might be related to the differences in the overall charge distribution of Pd/Cu-NPs vs. Pd/Cu(111). There are various counteracting forces that might be responsible for Pd dispersion vs. agglomeration, e.g. repulsive interactions between identically charged atoms (partly negatively charged Pd) and thermodynamic driving forces to build Pd-Pd or Pd-Cu bonds. With this, the overall stability of single atoms might critically depend on the subtle differences in the specific geometric configuration of the Cu host, e.g. on the number of immediate Cu neighbors interacting with Pd, which can affect both the charge distribution across the surface as well as the overall system energy. Indeed, it might be principally possible that the repulsive interactions between single Pd atoms prevail when these Pd atoms are integrated into low-coordinated sites available on Cu-NPs (note that negative charge on Pd must be compensated by a few surrounding Cu neighbors that should become partly positively charged). On the extended Cu(111) terraces, the charge distribution on Pd and surrounding Cu might be quite different due to the fact that the compensating positive charge can be distributed over a greater number of Cu atoms. This effect might potentially result in a strongly different electrostatic situation on the surface, which makes the repulsion less important than the thermodynamic drive force for agglomeration. These and other related hypotheses can be proven or disproven only at the theoretical level by calculating the related properties for suitable models, which are currently in planning and will be subject of forthcoming publications.

9. Applicability and limits of other experimental methods for addressing the structural properties of model SAA-based catalysts.

Additional structural characterization techniques can be employed for investigation of bimetallic alloys. Thus, Frenkel and his group recently published an excellent study, in which GI-EXAFS (performed in grazing incidence to approach surface sensitivity) was employed to address the structural properties of Pd-Ag and Pd-Cr-Ag alloys prepared as polycrystalline thin films under UHV conditions.[38] In this study, in which the state-of-the-art instrument was used, the authors investigated the alloys with relatively large Pd content (10 %), which appears to be close to the detection limit. Application of regular XAFS transmission mode, which probes the bulk and the surface together, was also reported for powdered SAA-based catalysts with formally lower concentrations of the active metal (a few per cent)[39], however, this content is related to the overall loading of the active metal in the entire particle, which might be significantly higher at the surface than in the bulk. In these types of studies employing regular XAFS, the information on the coordination situation of the active metal can be obtained only for the whole volume of the catalysts, including both bulk and the surface, and does not allow to judge specifically about the composition of the surface. It should be also noted that even if XAFS is performed in the grazing geometry, which is referred to as surface sensitive, the probing depth amounts to several layers (a few nm) of the catalyst, while the method developed in our study based on probing Pd sites by IRAS of CO is strictly sensitive only the top-most layer. Thus, XAFS provides valuable information on the structure of the nearest coordination spheres of the active atoms, however, it might be note very sensitive for highly diluted SAA-systems as investigated in this study and provides the information not only on the uppermost surface layer, but involves also several deeper lying layers of SAA.

ADF-STEM was so far successfully employed to image such systems as Au, Ag, Cu single atoms adsorbed on graphene[40] or single Pt atoms embedded in a monolayer MoS_2[41]. However, this method is highly challenging for imaging the SAA-based systems, as the contrast between the host and guest atom needs to be high enough and the atoms must be stable under the electron beam, which is harder to achieve for SAA-based systems. We are not aware of any studies, in which well-defined SAA-systems were investigated by ADF-STEM, but they would be certainly highly interesting.

REFERENCES

1. C. Schröder, P. A. Haugg, T. Görgens, S. Romaker, H. Gross, S. Schauermann, *Rev. Sci. Instrum.* **2025**, *96*, 015112.
2. E. Cisternas, F. Stavale, M. Flores, C. A. Achete, P. Vargas, *Phys. Rev. B* **2009**, *79*, 205431.
3. L. Padilla-Campos, A. Toro-Labbé, J. Maruani, *Surf. Sci.* **1997**, *385*, 24–36.
4. G. Kresse, J. Hafner, *Phys. Rev. B* **1994**, *49*, 14251–14269.
5. G. Kresse, J. Furthmüller, *Phys. Rev. B* **1996**, *54*, 11169–11186.
6. J. P. Perdew, K. Burke, M. Ernzerhof, *Phys. Rev. Lett.* **1996**, *77*, 3865–3868.
7. G. Kresse, D. Joubert, *Phys. Rev. B* **1999**, *59*, 1758–1775.
8. P. E. Blöchl, *Phys. Rev. B* **1994**, *50*, 17953–17979.
9. F. Viñes, F. Illas, K. M. Neyman, *Angew. Chem. Int. Ed.* **2007**, *46*, 7152–7155; *Angew. Chem.* **2007**, *119*, 7224–7227.
10. M. Bäumer, H.-J. Freund, *Prog. Surf. Sci.* **1999**, *61*, 127–198.
11. J. Libuda, F. Winkelmann, M. Bäumer, H. J. Freund, T. Bertrams, H. Neddermeyer, K. Müller, *Surf. Sci.* **1994**, *318*, 61–73.
12. A. E. Baber, H. L. Tierney, E. C. H. Sykes, *ACS Nano* **2010**, *4*, 1637–1645.
13. A. E. Baber, H. L. Tierney, T. J. Lawton, E. C. H. Sykes, *ChemCatChem* **2011**, *3*, 607–614.
14. G. Kyriakou, M. B. Boucher, A. D. Jewell, E. A. Lewis, T. J. Lawton, A. E. Baber, H. L. Tierney, M. Flytzani-Stephanopoulos, E. C. H. Sykes, *Science* **2012**, *335*, 1209–1212.
15. T. Wadayama, K. Kubo, T. Yamashita, T. Tanabe, A. Hatta, *J. Phys. Chem. B* **2003**, *107*, 3768–3773.
16. D. Zhang, V. Virchenko, C. Jansen, I. M. N. Groot, L. B. F. Juurlink, *J. Phys. Chem. C* **2025**, *129*, 3493–3497.
17. D. Zhang, C. Jansen, O. T. Berg, J. M. Bakker, J. Meyer, A. W. Kleyn, L. B. F. Juurlink, *J. Phys. Chem. C* **2022**, *126*, 13114–13121.
18. K. Horn, M. Hussain, J. Pritchard, *Surf. Sci.* **1977**, *63*, 244–253.
19. D. J. Stacchiola, *Acc. Chem. Res.* **2015**, *48*, 2151–2158.
20. K. Mudiyanselage, F. Xu, F. M. Hoffmann, J. Hrbek, I. Waluyo, J. A. Boscoboinik, D. J. Stacchiola, *Phys. Chem. Chem. Phys.* **2015**, *17*, 3032–3038.
21. K. Wolter, O. Seiferth, J. Libuda, H. Kuhlenbeck, M. Bäumer, H. J. Freund, *Surf. Sci.* **1998**, *402*, 428–432.
22. D. R. Rainer, C. Xu, P. M. Holmblad, D. W. Goodman, *J. Vac. Sci. Technol. A* **1997**, *15*, 1653–1662.
23. T. Wadayama, H. Osano, H. Yoshida, S. Oda, N. Todoroki, *Appl. Surf. Sci.* **2008**, *254*, 5380–5384.
24. C. M. Kruppe, J. D. Krooswyk, M. Trenary, *J. Phys. Chem. C* **2017**, *121*, 9361–9369.
25. D. A. Patel, R. T. Hannagan, P. L. Kress, A. C. Schilling, V. Çınar, E. C. H. Sykes, *J. Phys. Chem. C* **2019**, *123*, 28142–28147.
26. R. T. Hannagan, D. A. Patel, L. A. Cramer, A. C. Schilling, P. T. P. Ryan, A. M. Larson, V. Çınar, Y. Wang, T. A. Balema, E. C. H. Sykes, *ChemCatChem* **2020**, *12*, 488–493.

27. M. Muir, M. Trenary, *J. Phys. Chem. C* **2020**, *124*, 14722–14729.
28. J. Shi, C. J. Owen, H. T. Ngan, S. Qin, V. Mehar, P. Sautet, J. F. Weaver, *J. Chem. Phys.* **2021**, *154*, 234703.
29. D. L. Molina, M. Inagaki, E. Kazuma, Y. Kim, M. Trenary, *J. Phys. Chem. C* **2023**, *127*, 9796–9806.
30. A. Mohammadpour, S. Kaya, *J. Phys. Chem. C* **2024**, *128*, 5480–5489.
31. J. Wulfes, A.-K. Baumann, T. Melchert, C. Schröder, S. Schauermann, *Phys. Chem. Chem. Phys.* **2022**, *24*, 29480–29494.
32. J. Wulfes, A.-K. Baumann, M. Cieminski, C. Schröder, S. Schauermann, *J. Catal.* **2024**, *429*, 115213.
33. W. K. Kuhn, J. Szanyi, D. W. Goodman, *Surf. Sci.* **1992**, *274*, L611–L618.
34. J. Szanyi, W. K. Kuhn, D. W. Goodman, *J. Vac. Sci. Technol. A* **1993**, *11*, 1969–1974.
35. J. L. Davis, M. A. Barteau, *Surf. Sci.* **1990**, *235*, 235–248.
36. J. P. Camplin, E. M. McCash, *Surf. Sci.* **1996**, *360*, 229–241.
37. Z.-T. Wang, Y. Xu, M. El-Soda, F. R. Lucci, R. J. Madix, C. M. Friend, E. C. H. Sykes, *J. Phys. Chem. C* **2017**, *121*, 12800–12806.
38. J. F. Weaver, S. Xiang, J. Jamir, U. Küst, L. Rämisch, A. Grespi, H. Wallander, J. Zetterberg, S. Arias, E. L. Fornero, P. K. Routh, S. Zhang, J. S. Miller, R. K. Rai, E. A. Stach, J. A. Boscoboinik, M. M. Montemore, E. C. H. Sykes, J. Knudsen, J. Biener, L. R. Merte, A. I. Frenkel, *Angew. Chem. Int. Ed.* **2025**, e202513844.
39. T. Zhang, A. G. Walsh, J. Yu, P. Zhang, *Chem. Soc. Rev*. **2021**, *50*, 569–588.
40. M. Inazu, Y. Akada, T. Imaoka, Y. Hayashi, C. Takashima, H. Nakai, K. Yamamoto, *Nat. Commun*. **2022**, *13*, 2968.
41. H. Li, S. Wang, H. Sawada, G. G. D. Han, T. Samuels, C. S. Allen, A. I. Kirkland, J. C. Grossman, J. H. Warner, *ACS Nano* **2017**, *11*, 3392–3403.
